# A combined case of amelia and phocomelia in a neonate, at JFK Maternity Center, Liberia

**DOI:** 10.4314/gmj.v55i1.11

**Published:** 2021-03

**Authors:** Williams O Odunvbun, Billy C Johnson, Daniel W Kofa, Forkape B Duyenko, Magdalene E Odunvbun, Etedafe P Gharoro

**Affiliations:** 1 Department of Obstetrics and Gynaecology, John F. Kennedy Maternity Center, Monrovia. Liberia; 2 Department of Obstetrics and Gynaecology, College of Health Sciences, Delta State University, Abraka. Delta State. Nigeria; 3 Department of Paediatrics, JFK Memorial Hospital, Liberia; 4 College of Medical Sciences, University of Benin. Edo State. Nigeria

**Keywords:** Amelia, Phocomelia, Sporadic limb deformity

## Abstract

We report a case of spontaneous rare birth deformity. A case of Amelia and Phocomelia in a neonate. Amelia is a rare congenital disorder, even more so, is the combined amelia and phocomelia in a neonate. True Phocomelia was defined as the total absence of the intermediate segments of the limb. With the hand or foot (normal, almost normal, or malformed), directly attached to the trunk. The common aetiological association with phocomelia is from the use of thalidomide and genetic inheritance, as an autosomal recessive trait, involving chromosome 8. Isolated amelia is not generally considered to be of genetic origin. We present a neonate delivered by a 28-years multipara in Liberia, in West Africa Sub-Region, with amelia involving the two upper limbs, right lower limb and a Phocomelia involving the left lower limb (absence of tibia and fibula and feet with three toes). Africa is the only continent not included in the International Clearinghouse for Birth Defects Surveillance and Research. It is hoped that case reports of congenital limb deformities from Africa, will contribute to the formation of a database for birth defects shortly.

**Funding:**

None declared

## Introduction

Amelia is a congenital anomaly characterized by the complete absence of one or more limbs.^1^ Perhaps, the relatively more common limb deformity in the literature is Phocomelia. This is a rare congenital anomaly in which the proximal part of the limb (humerus or femur, radius or tibia, ulna, or fibula) is absent or markedly hypoplastic, with normal or nearly normal hand or foot^1^ The case we are presenting is a neonate with features of Amelia and Phocomelia

## Case Report

Ethical approval was obtained from the IRB Committee, JFK Maternity Centre #2021/01/jfk001.

This case was a neonate delivered by a registered 28-year-old Para 2,^+2^ with two previous live births. Both are females, healthy, and are growing normally. She had a history of 3 premarital surgical terminations of pregnancies between 6 weeks and 8 weeks.

The last one was about 10 years earlier. No known family history of congenital abnormalities. Consanguineous marital relationship is culturally forbidden in Liberia. She registered the pregnancy at a government health facility at 16 weeks.

There was no known history of ingestion of teratogenicmedication before index pregnancy. She had her previous two deliveries in the same facility. She neither smoked cigarettes nor indulged in other substances of abuse. Antenatal ultrasound scan that was requested at 20 weeks gestation was declined. She however presented at 38 weeks with 4 days history of liquor drainage, and 6 hours history of labour pains. Obstetric examination revealed a transverse lying foetus. She was having a uterine contraction of 3 in 10 minutes with moderate intensity. A quick speculum examination excluded cord prolapse, but the amniotic fluid was stained with fresh meconium.

An emergency room scan performed, confirmed the foetal lie.

**Figure 1:**
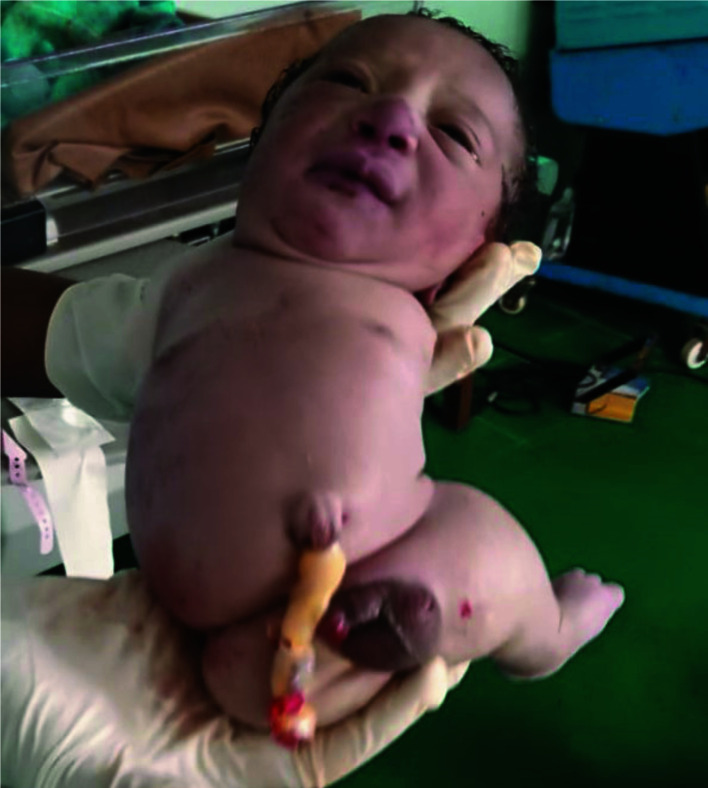
Male neonate with amelia of the two upper limbs and right lower limb.

The foetal heart rate was 110 beats per minute, there was a significant reduction in amniotic fluid. The foetal limb abnormalities were not observed. The patient subsequently had an emergency caesarean section for foetal distress. A live 2.0 kg male neonate with Amelia and Phocomelia was delivered (Figure 1). Apgar scores were 5 and 6 in the first and fifth minutes.

**Figure 2:**
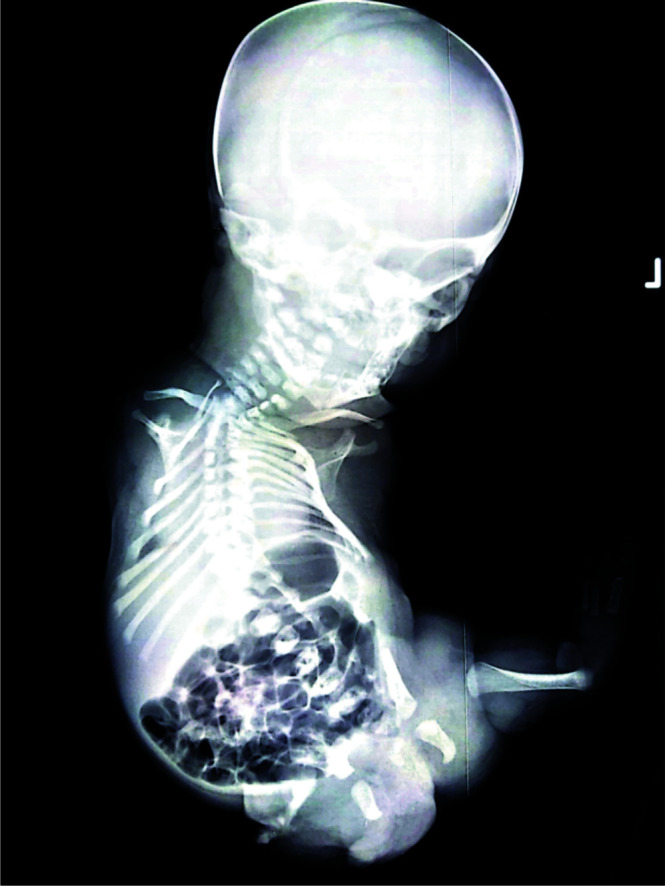
Post-mortem radiological image of the neonate confirming amelia of the upper limbs and right lower limb

**Figure 3:**
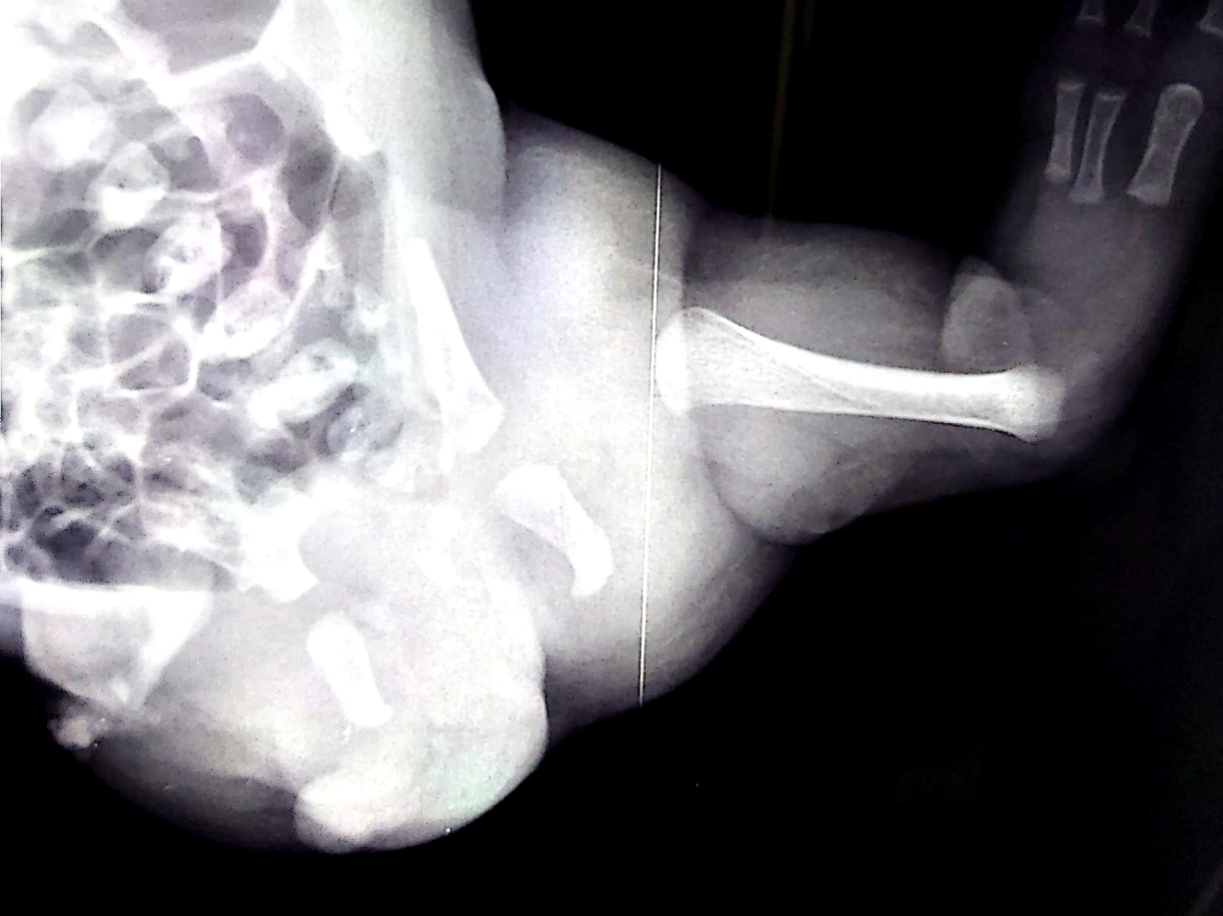
Radiological image confirming phocomelia of the left lower limb: Absence of tibia and fibula, with three toes

The baby was admitted to the neonatal intensive care unit for further resuscitation. The initial evaluation revealed respiratory distress, probably due to meconium aspiration in a neonate with limb deformities. There was no other gross abnormality on examination. Despite resuscitative measures, the neonate died 48 hours after delivery. The mother refused to see the baby in NICU. Informed consent was obtained for a post-mortem radiological evaluation of the baby (Figures 2 and 3). Consent for autopsy, for the evaluation of internal organs was declined.

## Discussion

The first case of phocomelia was described in Germany in 1956 in a baby whose mother had received thalidomide during pregnancy, in which the baby was born with only vestigial flipper-like hands without arms.^2^ Thalidomide was used as a sedative or hypnotic and was claimed to cure anxiety, depression, gastritis, and insomnia.^2^ Thalidomide has been effectively linked with widespread severe disabilities or death, among babies from Europe with utero deformities ranging from limb defects, deformed eyes, hearts, alimentary and urinary tract anomalies, blindness, and deafness^3^,^4^ Between 5000 and 7000 babies were born with phocomelia in Europe, of whom only 40% of babies whose mother took thalidomide survived in Germany.^5^ The drug was withdrawn from Germany in 1959.^2^ The mother of our patient had no history of thalidomide or any drug use, in the first trimester. Thalidomide is not available in Liberia.

Human limb development begins at about the 26^th^ day following fertilization, for the upper limb and the 28^th^ day, for the lower limb and completed at about the 56^th^ day.^1^ Primary ossification centers of the long bones of the limbs occurs by the 12^th^ week of development.^1^ The genetics of limb development is less understood. Some genes or gene families and molecular genetic factors are associated with the growth and differentiation of the developing limb.^6^

The case reported had amelia of three limbs and Phocomelia of one leg. Amelia is a rare congenital malformation. Among more than 23.1 million births from all over the world, the overall prevalence of amelia was 1.41 per 100,000 and ranged from a minimum of 0.42 to a maximum of 2.44.^1^ This case is the only one observed in this facility, with an average annual delivery rate of 3000, in the last three years. This gives an institutional prevalence of 11.1 per 100,000 deliveries.

A major reason for variation in the prevalence rate of amelia in the literature is classification.

Amelia is sometimes classified under other types of limb defects, such as transverse limb deficiencies. In many instances, amelia has been classified jointly, along with phocomelia.^7^ The case presented can justifiably be classified as amelia and phocomelia. There is perhaps a need for a class made up of neonates with features of both amelia and phocomelia like this case reported. Causes of limb defects include Sporadic phocomelia, environmental factors such as teratogens, a combination of genetic and environmental factors (multifactorial inheritance), vascular disruption, and ischaemia, as in limb reduction defects, Holt -Oram syndrome, thrombocytopenia- absent radius syndrome, and Robert's syndrome.^8^,^9^

Sporadic phocomelia is a genetic disorder inherited as an autosomal recessive trait or as spontaneous mutations.^5^ When both parents are carriers, there is a 25% chance of a child being affected. In cultural settings that encourage consanguineous marital relationships, the risk of phocomelia is expected to be high. Detail family history is needed to establish the presence of hereditary phocomelia. It is generally believed in Liberia, that the birth of a grossly malformed child outside public health facilities may not be documented. Such neonates are hardly accepted by parents, just like the case presented. It is plausible that this case resulted from spontaneous mutation. Holt-Oram syndrome can result from spontaneous genetic mutations or as an autosomal dominant disorder. The profile of our patients does not fit the features of Holt-Oram syndrome. The features of the syndrome include abnormal limb development that affects mostly the forearm and the carpal bones of the wrists, presenting like a hypoplastic thumb or a thumb that looks like a finger. The radius is usually missing and the humerus underdeveloped. The scapula and clavicle may also be affected. Seventy-five percent of patients with Holt-Oram syndrome have cardiac problems. This may include atrial septal defects or ventricular septal defects.^10^

Thrombocytopenia-absent radius syndrome (TAR) is characterized by a low platelet count, and absent radius, a hypoplastic thumb, and cardiac abnormalities.^11^ Bilaterally, both upper limbs were absent from our patient and there was no account of bleeding diathesis. Roberts Syndrome is a rare abnormality. Globally, only 150 cases have been reported. It is characterized by malformation of the bones in the face, skull, arms, and legs.^12^ Our patient does not have any facial deformities, such as cleft lip or palate, micrognathia, or hypertelorism, which often accompany the phocomelia in Roberts syndrome.

## Conclusion

It seems likely our patient's birth defect may well have resulted from spontaneous mutation. Diagnosis of most foetal anomalies can be made on antenatal scan at 18–20 weeks. This offers the parents the option of elective termination of the pregnancy.
